# Aggressive behavior, emotional, and attention problems across childhood and academic attainment at the end of primary school

**DOI:** 10.1007/s00127-021-02039-3

**Published:** 2021-02-22

**Authors:** Nathalie Tamayo Martinez, Henning Tiemeier, Maartje P. C. M. Luijk, James Law, Jan van der Ende, Frank Verhulst, Pauline W. Jansen

**Affiliations:** 1 The Generation R Study Group, Erasmus University Medical Center, Rotterdam, The Netherlands; 2 Department of Child and Adolescent Psychiatry/Psychology, Erasmus University Medical Center, Rotterdam, The Netherlands; 3 Department of Social and Behavioral Science, Harvard TH Chan School of Public Health, Boston, USA; 4 Department of Psychology, Education and Child Studies, Erasmus University Rotterdam, Rotterdam, The Netherlands; 5 School of Education, Communication and Language Sciences, Newcastle University, Newcastle-upon-Tyne, UK; 6 Child and Adolescent Mental Health Center, Mental Health Services, Capital Region of Denmark, Copenhagen, Denmark; 7 Faculty of Health and Medical Sciences, Department of Clinical Medicine, University of Copenhagen, Copenhagen, Denmark

**Keywords:** Academic attainment, Aggressive behavior, Emotional problems, Attention problems, Cohort study

## Abstract

**Purpose:**

To assess whether aggressive behavior and emotional problems from early childhood onwards are related to academic attainment at the end of primary education, and whether these associations are independent of attention problems.

**Methods:**

Data on 2546 children participating in a longitudinal birth cohort in Rotterdam were analyzed. Aggressive behavior, attention and emotional problems at ages 1½, 3, 5 and 10 years were assessed with the Child Behavior Checklist. Academic attainment at the end of primary school (12 years of age) was measured with the CITO test, a national Dutch academic test score.

**Results:**

Aggressive behavior from age 1½ to 10 years was negatively associated with academic attainment, but these associations attenuated to non-significance when accounting for comorbid attention problems. For emotional problems, first, only problems at 10 years were associated with poorer academic attainment. Yet, when accounting for attention problems, the association reversed: more emotional problems from 1½ to 10 years were associated with a better academic attainment. Attention problems at ages 1½ to 10 years were negatively associated with academic attainment, independent of comorbid emotional problems or aggressive behavior.

**Conclusions:**

Attention problems across childhood are related to a poorer academic attainment, while emotional problems predicted better academic attainment. Moreover, the relationship between aggressive behavior and academic attainment was explained by comorbid attention problems. Future research should determine the mechanisms through which attention problems and emotional problems affect academic attainment, to inform strategies for the promotion of better educational attainment.

**Supplementary Information:**

The online version contains supplementary material available at 10.1007/s00127-021-02039-3.

## Introduction

Behavioral and emotional problems are common in childhood, tend to be stable from early childhood onwards [1–4] and are linked to a wide range of poor outcomes along the life course, including adolescent and adult mental disorders, substance use, and social problems [5, 6]. Broadly, behavioral problems, encompass aggressive behavior (e.g., rule breaking, conduct problems, and aggression towards others) on the one hand, and attention problems on the other hand. Emotional problems mainly include depressive symptoms (e.g., sadness, worrying) and anxieties (e.g., perfectionism or tidiness). There is substantial evidence suggesting that behavioral and emotional problems coincide with difficulties at school. For instance, academic attainment is poorer when children have attention problems [7–11], or when there are behavioral or other emotional problems, although these findings are less consistent [12–14]. In the current paper, we examine the association of behavioral and emotional problems from early childhood onwards with academic attainment at the end of primary education to address limitations of the available literature, and to complement current knowledge.

Education is an important determinant of health [15]; research across different disciplines relates a higher education to better health among adults. However, there is a possibility of reverse causation, with health determining the educational level an individual can follow and attain [16]. One way to address this limitation is to evaluate the extent to which (mental) health before school entry is associated with later school attainment. While there is substantial evidence suggesting that behavioral and emotional problems co-occur with difficulties at school, only few longitudinal studies have examined whether and how the presence of behavioral and emotional problems before school entry impact upon academic attainment. Kremer et al*.* [14] and Turney and McLanahan [17] found that behavioral problems at 3 years of age predicted poorer academic skills later, whereas Flouri et al*.* reported that problems before age 5 years were associated with lower cognitive abilities in later childhood [18]. In contrast, Gray et al*.* [19] reported that aggressive behavior from ages 1–3 years was not associated with reading skills at 9 years, while attention problems were negatively associated with later reading ability. Importantly, these three studies did not account for the problems around the time the school functioning was measured. As such, it remains unclear whether associations were explained by the co-occurrence of behavioral or emotional problems, rather than reflecting a prospective association. Indeed, clinical studies in adolescence provided evidence that the diagnosis of a current episode of a mental disorder has a stronger negative association with academic functioning than former episodes of mental disorders [12, 20]. Whether this effect also holds in the general population for less severe symptoms in earlier childhood remains unknown. Moreover, the above mentioned contradictory findings regarding the association between behavioral problems and academic attainment [14, 19] might be explained by the fact that Gray et al*.* adjusted for attention problems, while others did not [13, 14, 18]. Likewise, emotional problems have been linked with a poorer academic attainment [14], but once adjusted for attention problems, the association has been noted to become nonsignificant [11, 17] or even became a positive one [12]. Together, these findings suggest that the association of aggressive behavior and emotional problems with academic attainment might (partly) be explained by attention problems, as has been suggested by some studies [11, 12, 17, 19, 21]. Additionally, the relationship between problems might be different depending on the sex of the child. As has been noted for adolescents, internalizing problems may affect girls’ but not boys’ academic attainment [12].

In the current study, we addressed three aims. First, we assessed if childhood aggressive behavior, emotional, and attention problems before school entry, are related to academic attainment at the end of primary education—independently of the problems during the primary school period. Second, we assessed if problems prior to school entry and across the primary school period are similarly related to academic attainment at the end of primary education. Third, we determined whether aggressive behavior and emotional problems before school entry and during the primary school period are related to academic attainment independently of attention problems.

## Materials and methods

### Participants

This study uses the data from Generation R, a population-based prospective cohort that enrolled 9778 pregnant women living in Rotterdam between April 2002 and January 2006. The study has been described extensively elsewhere [22]. Information on the mother, her partner and her child has been collected through questionnaires, hands-on measurements, and linkage to national databases, including a specific database on school attainment (CITO, see further details below). Out of 9 749 live births, 7 893 children participated in the preschool period, and 7 398 in the school period. Of the latter, 25.4% (*n* = 1 878) had missing information on academic attainment due to no consent to obtain information from the national database on school attainment, while among 39.2% (*n* = 2 886) of the children, another school attainment test than the CITO test was used by the schools. Furthermore, 88 participants were additionally excluded as no measurement of aggressive behavior, emotional *or* attention problems was available, leaving 2546 subjects for analyses (Supplementary Fig. 1). This study has been approved by the Medical Ethical Committee of the Erasmus Medical Centre, Rotterdam, and written informed consent was obtained from the parents of the participating children.

### Outcome: academic attainment

In the Netherlands, most children start primary education at age 4 years. It is mandatory from age 5 years onwards. In the final grade of primary school, when children are 11–12 years, it is mandatory to administer an academic test, which is used to guide the choice for secondary education (i.e., pre-vocational secondary education, higher general secondary education, and pre-university level). Of the different available academic tests, the CITO test is most frequently used. The test was developed by the Central Institute for Test Development (in Dutch: Centraal Instituut voor Test Ontwikkeling, CITO) [23]. The test evaluates academic attainment at the end of primary education by assessing language and mathematics skills. The CITO test was performed at a mean age of 12 years (SD = 0.4). The test score reflects a standardized score, ranging between 500 and 550, with higher scores meaning higher academic attainment. We transformed these scores to standardized scores with a mean of 0 and a standard deviation of 1.

### Exposures: aggressive behavior, emotional and attention problem scores

Aggressive behavior, emotional, and attention problems were measured using the parent rated aggressive behavior, internalizing and attention problem scales from the Child Behavior Checklist (CBCL) [24]. The CBCL is a widely used inventory that is a reliable and validated screening tool for mental health in children [25], which has been shown to predict DSM-based psychiatric disorders [26]. They were assessed four times: three times using the preschool form at 1½, 3, and 5 years (CBCL/1½-5) and once with the school form at 10 years (CBCL/6–18). The different versions measure the same underlying constructs using age-appropriate items. The aggressive behavior scale consists of 19 items in the CBCL/1½–5 form and of 18 items in the CBCL/6–18 form, with 9 overlapping items (e.g., “Gets in many fights”). We deliberately choose to not use the externalizing scale of the CBCL, as in the CBCL/1½–5, the externalizing scale incorporates the attention problems domain, while we aimed to study the specific role of attention problems. The CBCL/1½–5 internalizing scale consists of 36 items and includes the emotionally reactive (“Worries”), anxious/depressed (“Excessively tidy or too clean”; “Feels he/she has to be perfect”), somatic complaints (“Aches or pains”), and withdrawn scales (“Withdrawn, doesnot get involved with others”). The CBCL/6–18 internalizing scale consists of 32 items (11 overlapping) and includes the anxious/depressed, withdrawn/depressed and somatic complaints scales. We further refer to the internalizing scale as “Emotional Problems”. The CBCL/1½–5 attention problems scale consists of five items, and the CBCL/6–18 attention problems scale consists of ten items, with two overlapping items (i.e., “Cannot concentrate, cannot pay attention for long”; “Cannot sit still, restless, or hyperactive”). At each age, items were scored on a three-point scale from “not true” (0) to “very true or often true” (2). Items of a scale were summed if no more than 25% of the items were missing*.* Higher scores indicate more problems. The scores at 10 years were weighted to have the same range as the previous assessments. Therefore, the scores ranged at the different ages for the aggressive behavior scale from 0 to 36, for the internalizing scale from 0 to 72 and for attention problems scale from 0 to 10. The Cronbach’s alpha ranged from 0.82 to 0.87 for the internalizing scale, from 0.86 to 0.89 for the aggression scale and from 0.65 to 0.81 for the attention scale.

### Covariates

Covariates of the mother included age at enrolment during pregnancy, educational attainment, marital status, working status, and IQ. Educational attainment was categorized as: 3 years of secondary school or less (typically corresponds with 11 years of education); more than 3 years of secondary school or intermediate vocational training (generally corresponds with 12–15 years of education); higher vocational training (typically corresponds to 16 or 17 years of education) and university degree (usually indicates 18 years of education or more). At child age 5 years, marital status was assessed and classified as married/living together versus single parenthood, while mothers’ working status was defined as a paid job/studying vs. no paid work. Mother’s IQ was estimated with the set I from the Ravens Advanced Progressive Matrices Test [27], also at child age 5. Covariates of the child were sex, national origin, general health, and IQ. Child national origin was categorized as Dutch, Western, and non-Western, with a non-Dutch origin being assigned if one of the parents was born abroad. The Western category included those with a European or North-American origin. Those with a non-Western origin included Surinamese, Dutch Antillean, Turkish, African, and Asian descents. The child’s general health was reported by mothers at age 9 years using one item (“How would you describe the health of your child in general?”) with categories excellent, very good, good, and poor. The child’s IQ was assessed at 6 years using the validated Dutch Snijders–Oomen nonverbal intelligence test [28].

### Data analysis

**Fig. 1:**
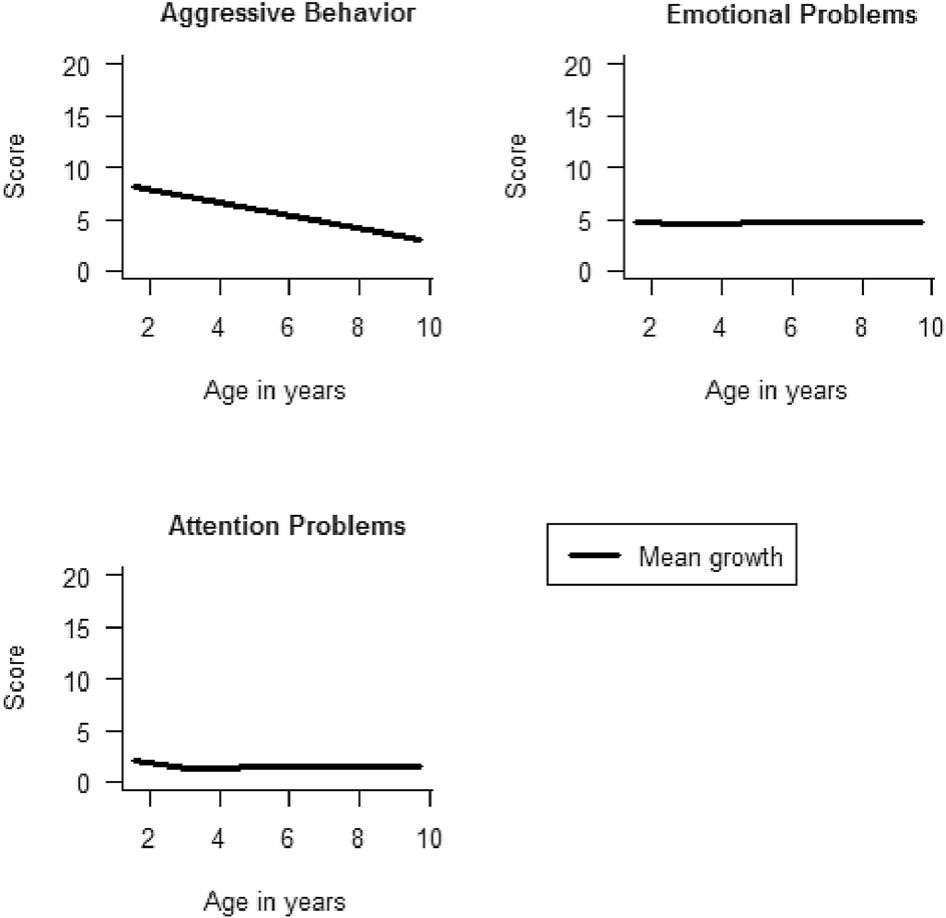
Estimated mean growth trajectories of problem scores from 1 ½ to 10 years. *Note.* Complete output presented in Supplementary Table 1

We checked correlations between the problem scores across ages. This study followed a two-step approach. The first step describes the mean change of the problem scores from age 1½ to 10 years and is needed to answer the study objectives in the second step. In the first step, we created for each participant growth curves of the aggressive behavior, emotional, and attention problems with the scale scores assessed at ages 1½, 3, 5, and 10 years, using linear mixed models (LMM). For each scale, we created different models, where the intercept was set at one of the different ages at which the assessments took place [29, 30]. The age of the child at each assessment was set as a continuous variable, and the models accounted for the correlation between the repeated measurements. Models were fitted with the restricted maximum likelihood estimation. To find the best fitting slope, we tested quadratic and cubic terms in the models. In the model, the fixed effect intercepts represent the calculated mean score at that age. The fixed effect slopes represent mean change of the problems across time, also called trajectory (Fig. 1 and Supplementary Table 1). The random effect intercepts and slopes represent the intercept and slope for each child, these were standardized to a mean of 0 and a standard deviation of 1 for the analysis in the second step.

**Table 1:** Characteristics of the study population

	Included participants (*N* = 2546)
Gender, % boy	47.5
Age at academic test (in years), Mean, SD	11.9 (0.4)
Academic test (score), mean, SD	538.5 (9.4)
Age mother at intake (in years), Mean, SD	31.4 (4.6)
Child IQ (score), mean, SD	104.4 (15.1)
Mother IQ (score), mean, SD	95.8 (15.0)
National origin of the child, %	
Dutch	67.3
Western	8.5
Non-Western	24.2
Child’s health, %	
Excellent	40.1
Very good	36.0
Good to poor	23.9
Maternal education, %	
3 years of secondary school or less	7.8
More than 3 years of secondary school	27.7
Higher vocational training	30.3
University degree	34.2
Single motherhood at child’s age 5 years, %	11.2
Maternal working status, %	
Working or studying	83.5
Unemployed	4.2
Housewife	12.3

In the second step, linear regression analyses were conducted, with academic attainment as the outcome and the intercepts from the first step as exposures. Covariates included in these analyses were the slopes from the first step, and the covariates listed above. Specifically, to assess the first objective, if problems from a very early age are related to academic attainment at the end of primary education independently of problems at later ages, the exposure was the intercept of problems set at 1½ and 3 years. We also evaluated if these associations depended on the sex of the child by testing sex interactions. For the second objective, to address if preschool aggressive behavior, and emotional and attention problems are as strongly related to academic attainment as problems at later ages, a *z* test was used. We compared the intercept coefficients of the different linear regression models which were set at different ages (1 ½, 3, or 10 years) [31].

To answer the third objective, to determine whether aggressive behavior and emotional problems across childhood predict academic attainment independently of attention problems, we repeated the linear regression analyses of the first and second objectives, with academic attainment as the outcome and aggressive behavior or emotional problems intercepts as exposures, while including the intercept and slope of attention problems as additional covariates.

Finally, three sensitivity analyses were conducted. First, to check whether associations were not solely driven by children who have higher problem scores, we repeated the analyses after excluding the children who scored above the 90th percentile on the problem scores at any age from 1½ to 10 years. Second, if child IQ is a confounder, adjusting for it is the correct approach. But child IQ can also be a mediator meaning that models would be over-adjusted if we included it as a covariate. To give insights into the influence of IQ on the models, we repeated the analyses without child IQ as covariate. Third, we corrected for potential selection bias due to children being lost to follow-up, using inverse-probability weighting. For this, a logistic regression model was fitted to predict the probability of attrition, from which an inverse-probability-of-attrition weight was computed for each participant. We re-ran the models for objective 1–3 weighting for the inverse probability of attrition [32].

The statistical program R 3.5.1 was used. For the LMM, the package Linear and Nonlinear Mixed Effects Models (nlme) was used [33]. The percentage of missing data in the aggressive behavior, emotional problems, and the attention problems score variables ranged from 8.1 to 28.7% and in the covariates from 0.6 to 26.9%. Missing values of the covariates were imputed by multiple imputation using chained equations (with package MICE) [34]. Fifty imputed data sets were created and the presented results are the estimates averaged across these fifty data sets. Academic attainment and CBCL problem scores were not imputed with MICE; for the CBCL problem scores, LMM imputes the missing data automatically.

## Results

### Non-response analysis

A non-response analysis indicated that the children included in the study (*n* = 2 546) had a higher IQ, 104 (SD = 15.1) than the children with missing data (*n* = 4 852) (IQ = 99.2, SD = 15.2). The mothers of the included children in the study were highly educated (34.0% university degree) and less often single parent (11.2%) than the mothers of excluded children (27.5% university degree; 15.2% single parenthood). Additionally, children excluded from the analysis due to missing data on the academic attainment score tended to have slightly higher problem scores at all ages, with aggressive behavior on average being 0.25 to 0.81 points higher, emotional problems being 0.50 to 1.03 points higher, and attention problems being 0.13 to 0.26 points higher as compared to included children.

### Description of the population

The characteristics of the children included in this analysis are presented in Table 1. The cohort included 47.5% boys. In total, 67.3% of the children were Dutch, 8.5% Western, and 24.2% non-Western.

The Pearson correlation coefficients between the different problem scores were low to high (0.13–0.69), with the highest correlations occurring between different problem scales assessed at the same age (0.37–0.69). Moderate correlations were found for repeated assessments of the same scale between ages 1½ and 10 years: for aggressive behavior, correlations ranged from 0.56 to 0.58, for emotional problems from 0.49 to 0.53 and for attention problems from 0.54 to 0.57.

The growth-curve models from the first step of the analysis are shown in Fig. 1 (estimates are presented in Supplementary Table 1). These models indicated that overall, there was a decrease in aggressive behavior from age 1½ to 10 years, while emotional problems and attention problems remained stable in the same time period. The significant cubic terms in the modelling of emotional and attention problems were very close to 0. Therefore, the shape of these graphs is almost linear. For the second step of the analyses (i.e., objectives 1–3), the *random* effect intercepts and slopes created from LMMs were incorporated in the linear regression models as exposures. However, we only incorporated the linear terms for the problems in these analyses, because the nonlinear terms were not significantly associated with academic attainment (all *p* > 0.05), nor added information to the models (all Wald test *p* > 0.05), while there was a high correlation between the linear slope, quadratic, and cubic slope terms (> 0.9).

### Objective 1: Are aggressive behavior, emotional and attention problems before school entry related to academic attainment at the end of primary education?

**Fig. 2:**
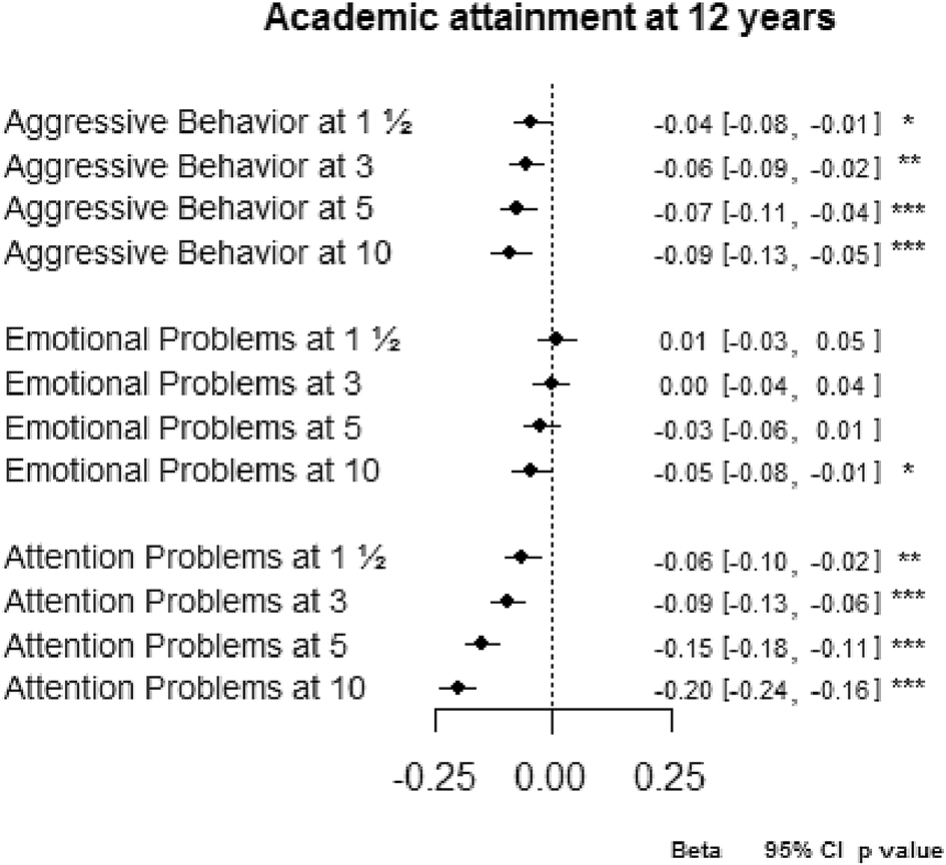
Relation between intercepts (problem scores) at different ages and academic attainment. Complete output presented in Supplementary Table 2. Each intercept represents one regression model. All models adjusted for the corresponding trajectories (slopes), and for maternal education, working status, single parenthood, and IQ, and for child gender, ethnicity, age at CITO assessment, general health and IQ. *p < 0.05, **p < 0.01, ***p < 0.001

Results of the analyses with the intercepts of the growth models set at the preschool ages are presented in Fig. 2 (slopes are presented in Supplementary table 2). For aggressive behavior, there was a significant association with educational attainment for problems at 1½ years and 3 years. A 1 SD increase in the aggressive behavior score at 1½ years reduced the academic attainment score by − 0.04 SD (95% CI − 0.08, − 0.01), when taking into account the trajectory of the problems. The results for the intercept at age 3 was similar. This means that aggressive behavior problems at an early age predict a lower school attainment independently of whether the problems change over time. For emotional problems, there was no significant association of the score at 1½ or 3 years with later school attainment (e.g., intercept at 1½ years = 0.01, 95% CI − 0.03, 0.05), when taking into account the trajectory of the problems. For attention problems, one SD increase in problems at 1½ or 3 years reduced the academic attainment with − 0.06 and − 0.20 SD (95% CIs − 0.10, − 0.02, and − 0.24, − 0.16), respectively, independently of whether symptoms change over time. Additionally, we found no interaction with child sex in the association of aggressive behavior, emotional and attention problems with academic attainment (data not presented).

### Objective 2: Are aggressive behavior, emotional and attention problems prior to and after school entry similarly related to academic attainment at the end of primary education?

To determine whether problems present in closer temporal proximity to academic attainment were more strongly related to academic attainment than earlier problems, we tested the linear models with the intercepts set at different ages (Fig. 2, Supplementary table 2). For all problem scales, the problems at an older age tended to be a stronger negative predictor of school attainment than the earlier problems. Although only the attention problems reached statistical significance (*p*s < 0.001).

### Objective 3: Are aggressive behavior and emotional problems associated with academic attainment independently of attention problems?

**Fig. 3:**
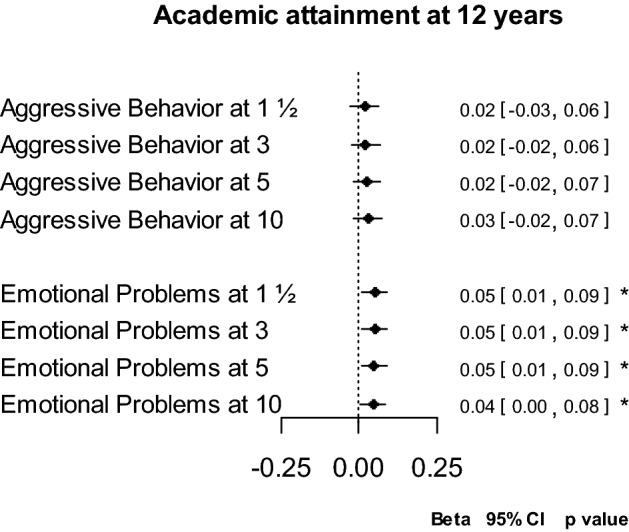
Relation between intercepts (problem scores) at different ages and academic attainment adjusting for attention problems. Output from Supplementary Table 3. Each intercept represents one regression model. All models adjusted for the corresponding trajectories (slopes), and for working status, single parenthood, maternal education and IQ, and child attention problems (intercept and slope at corresponding ages), gender, ethnicity, age at CITO assessment, general health at 10 years and IQ. *p < 0.05, **p < 0.01, ***p < 0.001

We then analyzed the association between aggressive behavior (and emotional problems) and academic attainment while adjusting for attention problems (see Fig. 3; slopes are presented in Supplementary table 3). When attention problems are taken into account, the results changed fundamentally. For aggressive behavior, the intercepts—whether set at 1½, 3, 5 or 10 years—were no longer significantly associated with academic attainment. Moreover, the associations of aggressive behavior at ages 1½ and 3 years with later academic attainment were not significantly different from the association of aggressive behavior at age 10 with academic attainment (*z* tests = 0.20 and 0.15, *p* values = 0.83 and 0.85, respectively).

For emotional problems, the intercepts at each age became significant positive predictors when adjusting for attention problems: more emotional problems were associated with a better school attainment. Additionally, when adjusted for attention problems, the intercepts for emotional problems at ages 1½ and 3 predicted academic attainment similarly as the intercept at age 10 (*z* tests = − 0.14 and − 0.71, *p* values = 0.88 and 0.86, respectively).

In the models evaluating the association of aggressive behavior and attention problems with academic attainment and in the models evaluating the association of emotional and attention problems with academic attainment (Supplementary table 3), the intercepts (and slopes) of attention problems at all ages remained significantly associated with academic attainment. This pattern was similar to the models including attention problems only, as presented in Fig. 2. Yet, attention problems at 10 years were more strongly related to a poorer academic outcome than the problems at 1½ and 3 years (*z* tests = − 4.11 and − 3.35, both *p* values < 0.001, respectively).

### Sensitivity analysis

The analyses were repeated after excluding the children who scored above the 90th percentile of any problem scale at any of the assessment ages (308 and 298 subjects excluded because of a high score on aggressive behavior and attention problems, or on emotional and attention problems, respectively). Results were similar as above, indicating that the associations of aggressive behavior, emotional, and attention problems with academic attainment affect children across the spectrum of problems and that the associations were not solely driven by children who have high scores. Likewise, if analyses were not adjusted for child IQ, results remain the same, suggesting that adding IQ to the model does not result in over adjustment. Finally, when correcting for potential selection bias with the inverse probability weights, results remain the same, except that in the linear models examining the first and second objectives, the intercept of emotional problems at 10 years becomes non-significant.

## Discussion

In a population-based prospective cohort of children, we studied the association of aggressive behavior, emotional, and attention problems before school entry with academic attainment at the end of primary education. Our findings suggest that attention problem substantially explained the associations of aggressive behavior and emotional problems with poorer academic attainment.

First, we found that aggressive behavior at ages 3, 5, and 10 years were associated with a relatively poor academic attainment. However, these associations were explained by attention problems. This suggests that the previously reported association between aggressive behavior and poor academic attainment [14] may largely be driven by the commonly comorbid attention symptoms. Although this has been suggested before for school age children [11], it has not been explicitly demonstrated in children as young as in the present study [19].

For emotional problems, first, we also found that more problems at 10 years of age were related to a poorer academic attainment 2 years later. However, once accounting for attention problems, emotional problems from 1½ to 10 years became positive predictors of later academic attainment. This suggests that in the absence of attention problems, children exhibiting some of the emotions or behaviors assessed in the emotional problems scale may somehow benefit either from these specified characteristics (e.g., perfectionism) or from broader characteristics they might represent (e.g., conscientiousness). Yet, because the comorbidity between emotional and attentional problems is moderate (0.37–0.44), this positive effect is only seen in a proportion of the children with emotional problems. Notably, although the effect sizes were small, this effect was similar regardless of the age at which emotional problems were assessed. These findings contrast with the previous studies finding either no association [11] or a negative association [17]. We are aware of only two papers with comparable results among adolescents. Veldman and colleagues [12] reported that the children with emotional problems at 11 years were more likely to attain a higher degree level in secondary education as compared to those without emotional problems, also when controlling for attention problems. Minkkinen and colleagues [35] found that 7th grade adolescents with emotional problems had a better academic attainment at the end of secondary education than the adolescents without emotional problems. We hypothesize that, in the absence of attention problems, certain items from the emotional problems scale, like “Too concerned with neatness or cleanliness”, “Self-conscious or easily embarrassed” or “Feels he/she has to be perfect” may lead to a better school achievement because it may motivate children to work hard at school. Indeed, in a study among adolescents with ADHD, those who reported mildly elevated trait anxiety performed better in cognitive tasks then adolescents without these traits [36]. On the contrary, classical symptoms of depression, such as lack of energy, sleep problems, and feelings of hopelessness, would hardly be positively associated with academic attainment. Contrary to our expectations and to previous literature showing that internalizing problems affected adolescent girls’ but not boys’ academic attainment [12], we did not find that child sex modified associations between problems and school achievement. Our finding might be related to the different raters of the problems; in the study mentioned above, the problems were rated by youth themselves who were also older than the children included in our study. This can also suggest that the association and its sex specificity might become more important in adolescence.

For attention problems, we found that the occurrence of symptoms from a very early age onwards were negatively associated with academic attainment, which was independent of children’s nonverbal intelligence. We also observed a graded relationship with problems closer to the academic evaluation being associated more strongly with poorer outcomes at school. This is in line with previous research, and has been the most consistent finding regarding behavioral and emotional problems and academic attainment in the literature so far [9, 11, 19]. We hypothesize that attention problems hamper the ability to choose and concentrate on relevant stimuli, which directly interferes with academic tasks and affects the learning process. Our findings highlight the need for interventions to improve children’s attention capacities and related skills to expand their learning potential and hence their academic attainment. Future research should be aimed at getting a better understanding of how attention problems negatively affect academic attainment, and whether and how emotional problems might have a beneficial effect on academic performance.

### Strengths and limitations of the analysis

The strengths of this analysis are the prospective nature of the data collection and multiple measurements over time, which allows to investigate the temporal relationship between the problems and academic attainment. The large number of children drawn from the general population, which makes the results generalizable to similar populations, amongst the characteristics found in this population was the low count of problems. Also, the outcome is a standardized test acquired through a national database, thus, avoiding recall bias, as well as minimizing bias that arises when the same person reports on the exposure and the outcome. A limitation of our analyses is that the ages at which the measurements were done, cover different developmental periods across childhood. Although the CBCL assessment at 10 years includes slightly different items than the CBCL preschool form which was used in our first three assessments. We solved this using the standardized scores, as the different versions measure the same underlying constructs. Indeed, the correlation between the CBCL’s aggressive behavior, emotional, and attention problems scores at 5 years and at 10 years had a similar magnitude as the correlation between the CBCL scores at 1½, 3, and 5 years, despite the larger time laps and different items assessed. The second limitation is that we used the aggressive behavior rather than the externalizing scale. For the preschool form, the externalizing scale consists of both aggressive behavior and attention problems, while we aimed to separate the specific effects of attention. To keep the measures consistent, we used the aggressive behavior scale that is available in both forms. Furthermore, we relied on mother reports on the CBCL only as there are no repeated assessments for other informants. Applying a multi-informant approach may have resulted in a more balanced evaluation of children’s problems. In particular for the assessed emotional symptoms, reporter bias is conceivable. Certain items in the emotional problem scale, such as tidiness and perfectionism, might result in a better school achievement. Yet, mother reports on such characteristics may also have been driven by high achievements of children, as by their own personal characteristics and expectations. As a result, the magnitude of the association between emotional problems and academic attainment may have been overestimated. Additionally, we missed information on the severity of problems, as we only had very limited diagnostic data on multiple mental disorders. Therefore, we cannot verify whether results are similar among children with a clinical diagnosis. Finally, the study population had a higher socioeconomic background than the original sample and the general population in the city of Rotterdam, which may limit generalizability of the results. Moreover, children lost to follow-up tended to have slightly more emotional and behavioral problems. This loss to follow-up may thus, have resulted in a lower statistical power to detect significant differences. However, our sensitivity analysis weighting the linear regression models for the probability of attrition bias provided similar results.

## Conclusions

Despite these limitations, our analyses point at a negative effect of attention problems and, in the absence of attention problems, a positive effect of emotional problems on academic attainment at a population level, where most of the children have only few symptoms. Although the effects were modest, at a population level, small effects may reflect larger individual differences. Additionally, these results add evidence to the reverse causality hypothesis suggesting that education not only affects health, but that health has a positive impact on educational attainment as well.

## Availability of data and material

The datasets generated and/or analyzed for the current study are not publicly available due to legal and ethical regulations, but may be made available upon request to data management of the Generation R Study (datamanagementgenr@erasmusmc.nl), in accordance with the local, national, and European Union regulations.

## Supplementary Information

Below is the link to the electronic supplementary material.

Supplementary file1 (DOCX 82 KB)

## Data Availability

The R studio codes are available upon request.
