# Supplementary material for: Aggressive behavior, emotional, and attention problems across childhood and academic attainment at the end of primary school
Source: Soc Psychiatry Psychiatr Epidemiol. 2021 Feb 22;56(5):837–46. doi: 10.1007/s00127-021-02039-3 (PMC8068650; doi:10.1007/s00127-021-02039-3)
Supplement: Supplementary file 1 — Supplementary file1 (DOCX 82 KB) [file 127_2021_2039_MOESM1_ESM.docx]

**Supplementary material**

Supplementary Table 1. Growth curve models representing the estimated mean change of Aggressive Behavior, Emotional and Attention Problems from age 1 ½ to 10 years (graphically depicted in Figure 1)

|  | **N** | **Beta for Problem scores** | 2.5%, 97.5% CI | p-value | Standard deviation of random effect | 2.5%, 97.5% CI |
| --- | --- | --- | --- | --- | --- | --- |
|  | *2 546* | *Aggressive Behavior* |  |  |  |  |
| Intercept at 1½ |  | 8.12 | (7.94, 8.30) | <.001 | 3.24 | (3.12, 3.37) |
| Slope linear |  | -0.64 | (-0.66, -0.61) | <.001 | <.001 | (<.001, 1.86) |
|  |  |  |  |  |  |  |
|  | *2 546* | *Emotional Problems* |  |  |  |  |
| Intercept at 1½ |  | 4.77 | (4.57, 4.97) | <.001 | 2.98 | (2.85, 3.15) |
| Slope linear |  | -0.25 | (-0.48, -0.02) | 0.04 | 0.39 | (0.35, 0.42) |
| Slope quadratic |  | 0.14 | (0.06, 0.21) | <.001 | <.001 | (<.001, 0.72) |
| Slope cubic |  | -0.01 | (-0.02, -0.01) | <.001 | 0.002 | (0.88, 1.05) |
|  |  |  |  |  |  |  |
|  | *2 546* | *Attention Problems* |  |  |  |  |
| Intercept at 1½ |  | 2.09 | (2.02, 2.16) | <.001 | 0.99 | (0.97, 1.03) |
| Slope linear |  | -0.79 | (-0.87, -0.71) | <.001 | 0.08 | (0.06, 0.09) |
| Slope quadratic |  | 0.20 | (0.17, 0.22) | <.001 | <.001 | (<.001, 0.13) |
| Slope cubic |  | -0.01 | (-0.02, -0.01) | <.001 | <.001 | (0.11, 0.30) |
| The assessment at age 10 was weighted to have the same range score as the previous assessments.  To graphically display the coefficients in Figure 1, we used as time grid sequence the mean age at each assessment (1½, 3, 5 and 10 years) when needed.  Slopes represent the change in the estimated problem score for every one year increase from age 1 ½ to 10 years | | | | | | |

Supplementary Table 2. Association between aggressive behavior, emotional and attention problems at different ages and academic attainment (Intercepts depicted in Figure 2).

|  |  | **Academic attainment** | |  |  |
| --- | --- | --- | --- | --- | --- |
| Age in years | **N** | **Beta** | **2.5%, 97.5% CI** | p.value |  |
| *Aggressive Behavior* |  |  |  |  |  |
| 1½ |  |  |  |  |  |
| Intercept | *2 546* | -0.04 | (-0.08, -0.01) | 0.02 |  |
| Slope |  | -0.05 | (-0.09, -0.01) | 0.006 |  |
| 3 |  |  |  |  |  |
| Intercept | *2 546* | -0.06 | (-0.09, -0.02) | 0.002 |  |
| Slope |  | -0.05 | (-0.08, -0.01) | 0.006 |  |
| 5 |  |  |  |  |  |
| Intercept | *2 546* | -0.07 | (-0.11, -0.04) | <.001 |  |
| Slope |  | -0.04 | (-0.08, -0.01) | 0.02 |  |
| 10 |  |  |  |  |  |
| Intercept | *2 546* | -0.09 | (-0.13, -0.05) | <.001 |  |
| Slope |  | -0.04 | (-0.08, 0.002) | 0.06 |  |
|  |  |  |  |  |  |
| *Emotional Problems* |  |  |  |  |  |
| 1 ½ |  |  |  |  |  |
| Intercept | *2 546* | 0.01 | (-0.03, 0.05) | 0.67 |  |
| Slope |  | -0.05 | (-0.09, -0.01) | 0.007 |  |
| 3 |  |  |  |  |  |
| Intercept | *2 546* | 0.00 | (-0.04, 0.04) | 0.88 |  |
| Slope |  | -0.05 | (-0.08, -0.01) | 0.01 |  |
| 5 |  |  |  |  |  |
| Intercept | *2 546* | -0.03 | (-0.06, 0.01) | 0.17 |  |
| Slope |  | -0.03 | (-0.07, 0.002) | 0.07 |  |
| 10 |  |  |  |  |  |
| Intercept | *2 546* | -0.05 | (-0.08, -0.01) | 0.02 |  |
| Slope |  | -0.05 | (-0.08, -0.01) | 0.02 |  |
|  |  |  |  |  |  |
| *Attention Problems* |  |  |  |  |  |
| 1 ½ |  |  |  |  |  |
| Intercept | *2 546* | -0.06 | (-0.10, -0.02) | 0.001 |  |
| Slope |  | -0.14 | (-0.18, -0.10) | <.001 |  |
| 3 |  |  |  |  |  |
| Intercept | *2 546* | -0.09 | (-0.13, -0.06) | <.001 |  |
| Slope |  | -0.13 | (-0.16, -0.09) | <.001 |  |
| 5 |  |  |  |  |  |
| Intercept | *2 546* | -0.15 | (-0.18, -0.11) | <.001 |  |
| Slope |  | -0.11 | (-0.14, -0.07) | <.001 |  |
| 10 |  |  |  |  |  |
| Intercept | *2 546* | -0.20 | (-0.24, -0.16) | <.001 |  |
| Slope |  | -0.11 | (-0.15, -0.07) | <.001 |  |
| Adjacent intercept and slope rows are one model.  All models adjusted for the mother’s working status, marital status, education, IQ, BSI and financial difficulties and the child’s gender, general health and IQ. | | | | | |

Supplementary Table 3. Association between aggressive behavior and emotional problems at different ages and academic attainment, adjusting for attention problems (Intercepts depicted in Figure 3)

|  | **Academic attainment** | | | | | | | |
| --- | --- | --- | --- | --- | --- | --- | --- | --- |
| Age in years | **N** | **Beta** | **2.5%, 97.5% CI** | **p.value** |  | **Beta** | **2.5%, 97.5% CI** | **p.value** |
|  |  | *Aggressive Behavior* | | |  | *Attention Problems* | | |
| 1 ½ |  |  |  |  |  |  |  |  |
| **Intercept** | 2 546 | 0.02 | (-0.03, 0.07) | 0.41 |  | -0.08 | (-0.13, -0.03) | 0.003 |
| **Slope** |  | 0.01 | (-0.04, 0.05) | 0.79 |  | -0.14 | (-0.18, -0.10) | <.001 |
|  |  |  |  |  |  |  |  |  |
| 3 |  |  |  |  |  |  |  |  |
| **Intercept** | 2 546 | 0.02 | (-0.02, 0.07) | 0.36 |  | -0.11 | (-0.15, -0.06) | <.001 |
| **Slope** |  | 0.01 | (-0.03, 0.04) | 0.80 |  | -0.13 | (-0.17, -0.09) | <.001 |
| 5 |  |  |  |  |  |  |  |  |
| **Intercept** | 2 546 | 0.02 | (-0.02, 0.07) | 0.28 |  | -0.16 | (-0.21, -0.12) | <.001 |
| **Slope** |  | 0.01 | (-0.03, 0.05) | 0.70 |  | -0.11 | (-0.15, -0.07) | <.001 |
| 10 |  |  |  |  |  |  |  |  |
| **Intercept** | 2 546 | 0.03 | (-0.02, 0.07) | 0.27 |  | -0.22 | (-0.27, -0.17) | <.001 |
| **Slope** |  | 0.00 | (-0.05, 0.05) | 0.98 |  | -0.11 | (-0.15, -0.06) | <.001 |
|  |  |  |  |  |  |  |  |  |
|  |  | *Emotional Problems* | | |  | *Attention Problems* | | |
| 1 ½ |  |  |  |  |  |  |  |  |
| Intercept | 2 546 | 0.05 | (0.01, 0.09) | 0.03 |  | -0.09 | (-0.13, -0.04) | <.001 |
| **Slope** |  | 0.00 | (-0.04, 0.04) | 0.92 |  | -0.14 | (-0.18, -0.10) | <.001 |
| 3 |  |  |  |  |  |  |  |  |
| Intercept | 2 546 | 0.05 | (0.01, 0.09) | 0.02 |  | -0.12 | (-0.16, -0.08) | <.001 |
| **Slope** |  | 0.00 | (-0.04, 0.04) | 0.97 |  | -0.13 | (-0.17, -0.09) | <.001 |
| 5 |  |  |  |  |  |  |  |  |
| Intercept | 2 546 | 0.05 | (0.01, 0.09) | 0.02 |  | -0.17 | (-0.21, -0.13) | <.001 |
| **Slope** |  | 0.00 | (-0.04, 0.03) | 0.81 |  | -0.11 | (-0.14, -0.07) | <.001 |
| 10 |  |  |  |  |  |  |  |  |
| **Intercept** | 2 546 | 0.04 | (0.003, 0.09) | 0.04 |  | -0.22 | (-0.27, -0.18) | <.001 |
| **Slope** |  | -0.01 | (-0.05, 0.03) | 0.60 |  | -0.10 | (-0.14, -0.06) | <.001 |
| Adjacent intercept and slope rows are one model, and the attention intercept is set at the same age as the emotional or aggression intercept.  All models adjusted for working status, marital status, maternal education and IQ, and child gender, ethnicity, age at CITO assessment, general health at 10 years and IQ.  In **bold** were the significant variables without attention in the model | | | | | | | | |

**9 749** live births

**Preschool period**

7 893 participants

478 withdrawn

1 166 excluded

174 loss to follow up

38 died postnatal

**School period**

7 398 participants

4 dead

491 withdrawn / loss to follow up

**2 546** subjects

analysis

No information

1 878 without consent for data linkage

2 886 without CITO assessed at the school

88 additional missing data on all repeatedly assessed CBCLs

Supplementary Figure 1. Flowchart of the study population
